# The Cedar Project: high incidence of HCV infections in a longitudinal study of young Aboriginal people who use drugs in two Canadian cities

**DOI:** 10.1186/1471-2458-12-632

**Published:** 2012-08-09

**Authors:** Patricia M Spittal, Margo E Pearce, Negar Chavoshi, Wayne M Christian, Akm Moniruzzaman, Mary Teegee, Martin T Schechter

**Affiliations:** 1 Centre for Health Evaluation and Outcome Sciences, Vancouver, BC, Canada; 2 University of British Columbia, Vancouver, BC, Canada; 3 Splatsin Secwepemc Nation, Enderby, BC, Canada; 4 Simon Fraser University, Vancouver, BC, Canada; 5 Carrier Sekani Family Services, Prince George, BC, Canada; 6 St Paul’s Hospital, 620B −1081 Burrard St, Vancouver, BC V6Z 1Y6, Canada

## Abstract

**Background:**

Factors associated with HCV incidence among young Aboriginal people in Canada are still not well understood. We sought to estimate time to HCV infection and the relative hazard of risk factors associated HCV infection among young Aboriginal people who use injection drugs in two Canadian cities.

**Methods:**

The Cedar Project is a prospective cohort study involving young Aboriginal people in Vancouver and Prince George, British Columbia, who use illicit drugs. Participants’ venous blood samples were drawn and tested for HCV antibodies. Analysis was restricted to participants who use used injection drugs at enrolment or any of follow up visit. Cox proportional hazards regression was used to identify independent predictors of time to HCV seroconversion.

**Results:**

In total, 45 out of 148 participants seroconverted over the study period. Incidence of HCV infection was 26.3 per 100 person-years (95% Confidence Interval [CI]: 16.3, 46.1) among participants who reported using injection drugs for two years or less, 14.4 per 100 person-years (95% CI: 7.7, 28.9) among participants who had been using injection drugs for between two and five years, and 5.1 per 100 person-years (95% CI: 2.6,10.9) among participants who had been using injection drugs for over five years. Independent associations with HCV seroconversion were involvement in sex work in the last six months (Adjusted Hazard Ratio (AHR): 1.59; 95% CI: 1.05, 2.42) compared to no involvement, having been using injection drugs for less than two years (AHR: 4.14; 95% CI: 1.91, 8.94) and for between two and five years (AHR: 2.12; 95%CI: 0.94, 4.77) compared to over five years, daily cocaine injection in the last six months (AHR: 2.47; 95% CI: 1.51, 4.05) compared to less than daily, and sharing intravenous needles in the last six months (AHR: 2.56; 95% CI: 1.47, 4.49) compared to not sharing.

**Conclusions:**

This study contributes to the limited body of research addressing HCV infection among Aboriginal people in Canada. The HCV incidence rate among Cedar Project participants who were new initiates of injection drug use underscores an urgent need for HCV and injection prevention and safety strategies aimed at supporting young people surviving injection drug use and sex work in both cities. Young people must be afforded the opportunity to provide leadership and input in the development of prevention programming.

## Background

Increasing levels of HCV infection among young Aboriginal people are distressing for many Aboriginal communities and service providers, not only in small and large urban centres but also in rural settings where healthcare resources are limited [1,2]. This situation is exacerbated by the fact that the major consequences of HCV infection, including cirrhosis of the liver and hepatocellular cancer do not develop for many decades after initial infection [3]. Available data have demonstrated that Aboriginal people are not only disproportionately represented among HCV infected people in Canada but also underrepresented in community based treatment programs [4]. In British Columbia, it is estimated that incidence for HCV infection is twice as high among Aboriginal people compared to non-Aboriginal people [5] and that over half the population of Aboriginal people living with HIV are HCV co-infected, making the treatment challenges associated with being HCV and HIV co-infected far more complex [6]. Unfortunately, national HCV surveillance data for indigenous people in Canada is considered to be very limited largely because of under reporting and lack of consistent documentation of ethnic status between provinces [7,8]. Despite these limitations the Public Health Agency of Canada has identified that HCV incidence among Aboriginal people in Canada is 5 to 6 fold higher than among non-Aboriginal people [8]. The majority of these new infections can be attributed to injection drug use and the sharing of contaminated injection equipment [7,9].

There is also increasing evidence that HCV is disproportionately impacting Aboriginal young people. As Roy et al [10]. have suggested, recent onset of injection is a particularly vulnerable time for young people and the risk for HCV seroconversion is extremely high. In a previous study, we demonstrated that approximately 11% of our participants will transition to injection drug use per year; a rate that is twice as high as that found in a similar longitudinal investigation conducted in Montreal [11,12]. A number of prospective studies in Canada have examined HCV infection among people who use injection drugs. However, most have been limited by the fact that they are older cohorts and characterized by high HCV prevalence and small numbers of Aboriginal people, making it difficult to assess risk factors relevant for Aboriginal specific programming.

Concerns over the paucity of available data and the potential for explosive HIV and HCV epidemics among Aboriginal young people in other areas of the province similar to those reported in the mid-1990s in Vancouver’s downtown eastside prompted the initiation of a two city cohort study to address the specific HIV and HCV related vulnerabilities of Aboriginal young people who use illicit drugs. To our knowledge, this is the only longitudinal study of young and ‘at risk’ indigenous peoples of its kind in North America. Follow-up of the cohort allowed us to estimate the incidence of HCV infection among young people enrolled in the Cedar Project and identify risk factors associated with seroconversion among study participants.

## Methods

The Cedar Project is an ongoing prospective study of young Aboriginal people who use drugs in Vancouver and Prince George, Canada. Previous studies have described the methodology of the Cedar study in detail including the proportion of Aboriginal young people living in BC, participant recruitment and questionnaire design [13]. Briefly, young people who self identified as Aboriginal people were considered to be the descendants of the First Nation Peoples of North America and include Métis, Aboriginal, First Nations, Inuit and status and non-status Indians. Participants living primarily in the downtown areas of both cities were recruited through referral by health care providers, community outreach and word of mouth. Eligibility criteria for study entry included age 14 to 30 years and to have smoked or injected illicit drugs, aside from marijuana, in the month prior to enrolment. Saliva screens (Oral-screen, Avitar Onsite Diagnostics) were used to confirm drug use. All participants met with one First Nations study coordinator who explained procedures, sought informed consent and confirmed study eligibility. At enrolment, participants completed a detailed interviewer-administered questionnaire to elicit information on socio-demographic characteristics, non-injection and injection drug use, injection practices, sexual vulnerability and service utilization. Participants were followed-up every six months for subsequent interviews. Venous blood samples were drawn and tested for HIV and HCV-antibodies at each visit, and each participant had private interviews including pre- and post-test counselling with trained nurses. Participants were requested but not required to return for their HIV/HCV serostatus test results and given a twenty dollar stipend at each study visit.

Since they were established in 2010, we have enthusiastically embraced the Tri-Council Policy Statement: Ethical Conduct for Research Involving Humans, with particular attention to Section 9, which pertains to research involving Aboriginal participants. Our First Nations collaborators and investigators were involved in the conception, design and interpretation of this study and approved this manuscript for publication. The University of British Columbia/Providence Health Care Research Ethics Board has also approved the study.

### Outcome variables and covariates

The main outcome of interest in this study was HCV antibody seroconversion among participants who reported injection drug use between January 2003 and January 2009. We used algorithms for HCV serologic testing similar to those used in other studies conducted in this region [14]. In brief, AxSYM HCV version 3.0 (Abbott Laboratories, Chicago, Ill.) was used to screen all plasma samples. Negative samples did not undergo further testing. All positive samples underwent further testing with the recombinant Ortho HCV 3.0 ELISA test system (Ortho Clinical Diagnostic Inc., Rochester, NY). Samples that tested positive with both assays were classified as positive. Samples that tested positive by the AxSYM HCV test and negative by the Ortho HCV test were classified as negative. Because of the known association of the HCV virus with parenteral drug use, HCV incidence rates were estimated by three categories using the variable time since initiation of injection drug use. The variable was coded for relatively equal distribution between participants who had been injecting for <2 years (n = 40), those who had been injecting for 2–5 years (n = 40) and those who had been injecting for over 5 years (n = 66).

The following fixed dichotomous baseline covariates were used in the analysis: interviewed in Vancouver; sex; identifying as gay, lesbian, bisexual or two-spirited; having at least one parent who had attended residential school; ever having been placed into foster care; ever having attempted suicide; and ever having experienced sexual abuse. Sexual abuse was defined as any type of sexual activity into which participants had either been forced or coerced (including childhood sexual abuse, molestation, rape and sexual assault). Nonfixed (occurred in the past six months) covariates included age, being in a relationship, having lived on the streets for a period of three nights or more, having been in jail or prison overnight or longer, having been denied shelter because of drug use, having been involved in sex work, having any sexually transmitted infection, having been incarcerated overnight or longer, presence of antibodies to HIV, time since initiation of injection drug use, daily vs. less than daily use of injection cocaine, daily vs. less than daily use of injection opioids (including heroin, morphine, methadone, dilaudid or talwin and speedballs (combination of cocaine and heroin)), daily vs. less than daily use of injection methamphetamine, rig sharing, having overdosed, and inconsistent use of condoms during insertive sex (vaginal, anal) with casual, regular or client partners. Sexual assault was defined as having experienced sexual violence in the past six months. Sex work was defined as receiving money, shelter, food or drugs in exchange for sex. Sexual relationships that lasted less than three months were defined as casual sexual partners, more than three months were defined as regular partners, and clients were relationships where sex was exchanged for money, food, drugs or shelter.

The Cedar Project cohort includes 605 participants in total, however this study included only participants who reported injection drug use, were HCV negative at baseline, reported injection drug use and who returned for at least one of eight follow-up interviews up to December 2008 (n = 148). HCV incidence was calculated as incidence density. HCV incidence was calculated separately for groups including participants from Vancouver and Prince George, all participants together, and for participants who had been using injection drugs for less than two years, those who had been using injection drugs for between two and five years, and those who had been using injection drugs for five or more years. The date of seroconversion was estimated using the midpoint between the last negative and the first positive antibody test result. Cumulative incidence rates of HCV infection were calculated using Kaplan Meier methods. In these analyses time 0 was defined as the date of enrolment. We assessed the proportionality assumptions using the log minus log curve for the fixed covariates used in the model and observed no violations of assumptions. Participants who consistently remained HCV seronegative were considered to be right censored at the time of their most recent test result. Annual rates of HCV seroconversion were calculated with actuarial methods per 100 person years. Relative risks and 95% confidence intervals were obtained for risk factors of interest.

Cox proportional hazards regression were used to examine the independent effect of both fixed and time-dependent covariates on time to HCV seroconversion. Adjusted and unadjusted time dependent Cox regression models were used to identify risk associations with HCV seroconversion with Hazard Ratios and 95% confidence intervals were obtained for risk factors of interest. Backward stepwise Cox proportional hazards model included all variables that were statistically associated with HCV seroconversion at a *p* <0.10 significance level in unadjusted analyses. The adjusted model controlled for the significant covariates to determine which of those risk factors are independently associated with HCV seroconversion. All reported *p*-values are two-sided.

Overall, 605 (305 in Vancouver and 300 in Prince George) young Aboriginal people were recruited and screened for enrolment in the Cedar Project study. Among them, 292 (48%) were women and the mean age was 23.5 years (Standard Deviation (SD): 4.0). A total of 335 (55.4%) participants reported that they had used injection drugs before enrolment, and 42 (6.9%) reported that they had initiated injection drug use in the time after enrolment. The mean age of first having injected drugs was 20.2 years (SD: 4.6). Among the 377 participants who had injected drugs over the study period, 175 were HCV seronegative at time of their baseline interview. Out of 175 participants, 148 completed at least one follow-up visit, yielding a follow-up rate of 85%. In total, 148 participants were therefore eligible for inclusion in the HCV incidence analysis.

## Results

In total, 148 participants were eligible for inclusion in the analysis. Descriptive baseline statistics are provided in Table 1. The median age of participants at baseline was 23 years (standard deviation: 3.9), 53.4% were women and 57.4% were located in Vancouver. In addition, 11.5% identified as gay/lesbian/bisexual/transgender or two-spirited, 52.1% reported that they had ever been sexually abused and 68.2% had ever been taken from their biological parents into the child welfare system. In total, these 148 participants contributed 338.6 person years of observation over the study period. The mean length of follow-up time was 2.6 years (SD: 1.6) for all participants, 1.3 years (SD: 1.1) for those who became HCV positive and 3.2 years (SD: 1.5) for those who remained HCV negative. Table 2 demonstrates the incidence of HCV among Cedar Project participants overall, and among those who reported using injection drugs for 2 years or less, between 2-5 years and over 5 years. Overall, 45 participants seroconverted during the observation period yielding an incidence density of 11.6 cases per 100 person years (95% Confidence Interval (CI): 8.5%, 17.1%). Incidence of HCV infection was higher in Prince George than in Vancouver (14.8 vs. 9.6 per 100 person years), but this difference was not statistically significant (Log-rank test, p = 0.186). Incidence of HCV infection was 26.3 per 100 person-years (95% CI: 16.3, 46.1) among participants who reported using injection drugs for two years or less, 14.4 per 100 person-years (95% CI, 7.7, 28.9) among participants who had been using injection drugs for between two and five years, and 5.1 per 100 person-years (95% CI: 2.6,10.9) among participants who had been in using injection drugs for over five years.

**Table 1:** Unadjusted and adjusted* backward stepwise Cox regression models for vulnerabilities associated with HCV incidence among Cedar participants who used injection drugs (n = 148)

**Variable**	**Baseline^ n (%)**	**Unadjusted HR (95%CI)**	**P value**	**Adjusted HR (95%CI)**	**P value**
Location				-	
Prince George	63 (42.6)	1.48 (0.82, 2.66)	0.676		
Vancouver	85 (57.4)	1.0 (Reference)			
Sex					
Female	79 (53.4)	1.43 (0.79, 2.60)		-	
Male	69 (46.6)	1.0 (Reference)	0.240		
Age at enrolment	23.1** (3.9)	0.95 (0.87, 1.02)	0.161	-	-
Sexual identity					
Gay/bisexual/two-spirited	17 (11.5)	1.62 (0.72, 3.63)	0.242	-	-
Straight	131 (88.5)	1.0 (Reference)			
At least one parent in residential school				-	-
Yes	81 (54.7)	0.95 (0.53, 1.70)	0.850		
No/unsure	67 (45.3)	1.0 (Reference)			
Ever taken into foster care				-	-
Yes	101 (68.2)	1.29 (0.67, 2.51)	0.445		
No	47 (31.8)	1.0 (reference)			
Ever sexually abused					-
Yes	76 (52.1)	1.66 (0.91, 3.04)	0.098	-	
No/unsure	70 (47.9)	1.0 (reference)			
Sexual assault last 6 months				-	-
Yes	3 (2)	1.19 (0.37, 3.85)	0.770		
No	145 (98)	1.0 (reference)			
Relationship status last 6 months				-	-
Single	115 (77.8)	0.96 (0.64, 1.45)	0.856		
In a relationship	33 (22.2)	1.0 (Reference)			
Ever on streets for >3 nights			0.845	-	-
Yes	56 (38.1)	0.94 (0.52, 1.72)			
No	91 (61.9)	1.0 (reference)			
On street for > nights last 6 months					-
Yes	n/a	1.26 (0.83, 1.90)	0.275	-	
No		1.0 (reference)			
Ever been in jail prison					
Yes	97 (66)	1.11 (0.59, 2.09)	0.742	-	-
No	50 (34)	1.0 (reference)			
Been in jail or prison last 6 months				-	-
Yes	38 (25.7)	1.25 (0.83, 1.89)	0.291		
No	110 (74.3)	1.0 (reference)			
Ever attempted suicide					
Yes	66 (44.6)	0.67 (0.37, 1.23)	0.195	-	-
No	82 (55.4)	1.0 (reference)			
Number of lifetime sexual partners			0.530	-	-
>20	70 (48.6)	1.21 (0.67, 2.18)			
<20/refused	74 (51.4)	1.0 (reference)			
Ever involved in sex work				-	-
Yes	67 (45.6)	1.63 (0.90, 2.96)	0.107		
No	80 (54.4)	1.0 (reference)			
Involved in sex work last 6 months					
Yes	47 (31.8)	**1.71 (1.16, 2.53)**	**0.007**	**1.59 (1.05, 2.42)**	**0.030**
No	101 (68.2)	1.0 (reference)		1.0 (reference)	
Condom use for insertive sex with regular partner last 6 months				-	-
Not always	62 (41.9)	0.82 (0.51, 1.34)	0.437		
Always	86 (58.1)	1.0 (reference)			
Condom use for insertive sex with causal partner last 6 months				-	-
Not always	28 (18.9)	0.60 (0.24, 1.48)	0.268		
Always	120 (81.1)	1.0 (reference)			
Condom use for insertive sex with clients last 6 months				-	-
Not always	6 (4.1)	1.96 (0.61, 6.36)	0.260		
Always	142 (95.9)	1.0 (reference)			
Ever had a STI					
Yes	65 (43.9)	1.17 (0.65, 2.12)		-	-
No	83 (56.1)	1.0 (reference)	0.604		
Had a STI last 6 months					
Yes	16 (10.8)	0.94 (0.36, 2.50)	0.908	-	-
No	132 (89.2)	1.0 (reference)			
Ever overdose					
Yes	48 (32.4)	0.76 (0.42, 1.40)	0.382	-	-
No	100 (67.6)	1.0 (reference)			
Overdose last 6 months					
Yes	20 (13.5)	1.71 (0.82, 3.58)	0.152	-	-
No	128 (86.5)	1.0 (reference)			
Time since initiation of injection drug use^1^					
< 2 years	n/a	**4.57 (2.19, 9.51)**	**<0.001**	**4.14 (1.91, 8.94)**	**<0.001**
2-5 years		**2.43 (1.09, 5.45)**	**0.031**	**2.12 (0.94, 4.77)**	**0.070**
> 5 years		1.0 (reference)		1.0 (reference)	
Frequency of injection cocaine last 6 months					
Daily	16 (10.8)	**3.94 (2.49, 6.24)**	**<0.001**	**2.47 (1.51, 4.05)**	**<0.001**
< Daily/non-user	132 (89.2)	1.0 (reference)		1.0 (reference)	
Frequency of injection methamphetamine last 6 months				-	-
Daily	4 (2.7)	1.79 (0.90, 3.57)	0.099		
< Daily/non-user	144 (97.3)	1.0 (reference)			
Frequency of injection opiates last 6 months				-	-
Daily	27 (18.2)	**2.06 (1.43, 2.98)**	**<0.001**		
< Daily/non-user	125 (81.7)	1.0 (reference)			
Syringe sharing last 6 months					
Yes	19 (12.8)	**3.35 (2.09, 5.39)**	**<0.001**	**2.56 (1.47, 4.49)**	**0.001**
No	129 (87.2)	1.0 (reference)		1.0 (reference)	
Reuse of own syringes last 6 months				-	-
Yes	13 (9.1)	**2.17 (1.48, 3.19)**	**<0.001**		
No	131 (90.9)	1.0 (reference)			
Hard to find new syringes last 6 months				-	-
Yes	15 (10.1)	**2.16 (1.31, 3.57)**	**0.003**		
No	133 (89.9)	1.0 (reference)			
Need help injecting last 6 months				-	-
Yes	38 (25.7)	1.14 (0.61, 2.14)	0.687		
No	110 (74.3)	1.0 (reference)			
Accessed any alcohol or drug treatment program last 6 months				-	-
Yes	31 (21.1)	1.25 (0.81, 1.93)	0.317		
No	116 (78.9)	1.0 (reference)			
Unable to access drug treatment program				-	-
Yes	22 (15)	0.90 (0.48, 1.69)	0.741		
No	125 (85)	1.0 (reference)			
Currently in methadone treatment program				-	-
Yes	5 (3.4)	2.11 (0.83, 5.37)	0.117		
No	143 (96.6)	1.0 (reference)			

*All variables significant at the p ≤ 0.10 level in unadjusted analysis were included in the adjusted backward stepwise Cox proportional hazards model.

*HR* Hazard Ratio.

^Responses with less than n = 148 participants reflect missing data.

**Mean age (standard deviation).

^1^For those who had seroconverted to HCV positive, variable was time to seroconversion; and those who were non-incident cases, variable measured time to last follow up visit since initiation of injection.

n/a: not available.

**Table 2:** HCV incidence among Cedar Project participants who had been using injection drugs for less than 2 years, 2–5 years and over 5 years

**HCV incidence**	**Incident cases**	**Total person years**	**Incidence density (per 100 person years)**	**95% Confidence Interval (per 100 person years)**
Overall	45	387.9	11.6	8.5- 17.1
≤2 years since initiation of injection drug use	21	79.7	26.3	16.3-46.1
2-5 years since initiation of IDU	13	90.4	14.4	7.7-28.9
Over 5 years since initiation of IDU	11	214.5	5.1	2.6-10.9

**Figure 1:**
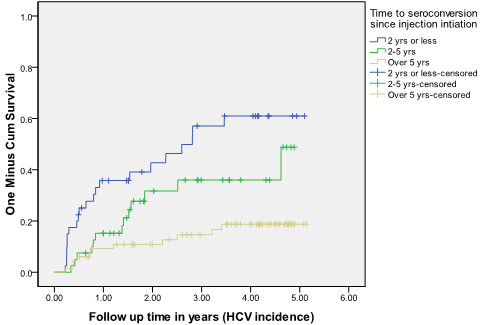
Kaplan Meier Curves of HCV incidence rates by length of time participants reported use of injection drugs (2 years or less, 2–5 years, and over 5 years).

Figure 1 demonstrates the survival estimates for time to HCV seroconversion among participants who had only been injecting for two years or less, those who had been using injection drugs for between two and five years, and those who had been injecting for over five years. For those newest to injection drug use, the cumulative HCV incidence increased sharply to approximately 38% within the first year of follow-up and was over 50% by the third year of follow-up.

Results from unadjusted and adjusted Cox regression analyses for factors associated with HCV incidence are displayed in Table 1. In unadjusted analyses, risk for HCV seroconversion was significantly associated with having been involved in sex work in the last six months (Unadjusted Hazard Ratio (UHR): 1.79; 95% CI: 1.16, 2.53). Risk for seroconversion appeared to decline with duration of injecting, as the hazard for HCV was significantly higher for participants who reported having been injecting drugs for less than two years (UHR: 4.57; 95% CI: 2.91, 9.51), and for those who had been injecting for between two and five years (UHR: 2.43; 95% CI: 1.09, 5.45) compared to those who had been injecting for five or more years. HCV risk also appeared to rise with increased frequency of injection, as participants who reported daily cocaine injection in the last six months (UHR: 3.94; 95% CI: 2.49, 6.24) and daily opioid injection in the last six months (UHR: 2.06; 95% CI: 1.43, 2.98) had significantly higher rates of HCV incidence compared to participants who were injecting cocaine or opioids less than daily. In addition, participants who reported unsafe injection practices in the last six months including sharing rigs (UHR: 3.35; 95% CI: 2.09, 5.39), reusing one’s own rig (UHR: 2.17; 95% CI: 1.48, 3.19) and having a difficult time finding new rigs (UHR: 2.16; 95% CI: 1.31, 3.57) had significantly higher risk for HCV seroconversion.

In the adjusted backward stepwise Cox regression model, independent associations with HCV seroconversion among Cedar Project participants who used injection drugs were involvement in sex work in the last six months (Adjusted Hazard Ratio (AHR): 1.59; 95% CI: 1.05, 2.42), having been using injection drugs for less than two years (AHR: 4.14; 95% CI: 1.91, 8.94) and for between two and five years (AHR: 2.12; 95%CI: 0.94-4.77), daily cocaine injection in the last six months (AHR: 2.47; 95% CI: 1.51, 4.05) and sharing rigs in the last six months (AHR: 2.56; 95% CI: 1.47, 4.49).

## Discussion

Our findings have illustrated that young Aboriginal people are at very high risk of HCV infection early in their injection careers. The declining risk with longer durations of injection was not surprising. It is likely that a form of survivorship bias is operative here. The analysis was based on those who were HCV negative at recruitment, therefore highest risk individuals in the longer-using groups would have seroconverted to HCV prior to the study, leaving lower risk groups for inclusion in this analysis. However, it is shocking that approximately 26% of participants became infected with HCV within two years of initiating injection drug use in two cities with established harm reduction programs. It is clear from this observation that the timeframe in which to provide access to meaningful preventative interventions after these young people initiate injection drug use is very short and that harm reduction programs must do a better job of reaching them [11,15,16].

This study also demonstrated that participants who reported frequent drug injection and syringe sharing over the study period had over twice the risk for HCV infection. Poly-drug use was common in this group. More than half of those who reported daily cocaine injection also reported daily opiate injection (data not shown). It is noteworthy that daily cocaine injection remained associated with HCV infection in the multivariable model while daily opiate injection did not. This is consistent with a previous study in Vancouver that demonstrated acquisition of HIV among injection drug users was directly related to frequent use of injection cocaine as opposed to opiates, particularly as a consequence of ‘cocaine binges’ [17]. We find it very concerning that despite scaling up harm reduction efforts, high intensity injection cocaine and syringe sharing continues to be exposing Cedar Project participants to high risk for HCV infection in both the northern and southern urban communities. Increased efforts must be made to engage young people who are new to injection drug use in harm reduction service provision, including injection equipment, addiction treatment and HIV and HCV testing services. Moreover, while early intervention with young people who have recently transitioned to injection drug use is immediately required, programming aimed at prevention of injection drug use must also be prioritized. In both Vancouver and Prince George, the complex interplay between the impact of colonization, residential schools, racialized care, and generations of mistrust of both provincial and federal authorities must be acknowledged [18]. When designing new programming service providers must be mindful of how the past can shape the response to prevention initiatives [19] and nurture flexible, trust-based relationships that seek to build upon young Aboriginal people’s resiliency in the face of intergenerational and lifetime traumas [20]. Any efforts to alleviate the immense impact of drug related harm but be inclusive of the perspectives of those young people who have learned how to ‘remain safe’ and have avoided infection via injection drug use [21]. Furthermore, young people must be afforded the opportunity to provide leadership in programme design intended to reflect their needs.

An independent association was also found in this study between recent involvement in sex work and HCV seroconversion. These results provide evidence that the risk for HCV among participants who had been involved in sex work was 59% higher compared to those who had not been involved in sex work. Recent studies conducted in British Columbia’s lower mainland have demonstrated that impoverished Aboriginal women involved in sex work and concomitant illicit drug use continue to be exposed to alarming levels of drug related harm, infectious disease and violent predation [22-24]. Sadly, these findings are consistent with other settings internationally where women exchanging sex for basic needs including drug dependence, are exposed to alarming levels of violence and infectious disease [25]. These data indicate the critical need for scaling up services aimed at reducing drug or sex related harm for young Aboriginal women, particularly in Northern and remote communities. It is important to highlight that many young Aboriginal women involved in sex work are intimately involved with men who are usually older and who also use injection drugs [23,26]. Indeed research has demonstrated that for women who rely on their intimate partners for drug acquisition, preparation and injection, the distribution of power and control in intimate relationships often lies with drug injecting men who control the money and the drugs [27,28]. Combined, these factors lead to a greater likelihood of unsafe sex and the female partner more likely coming “second on the needle.”

Several limitations of this study should be acknowledged. This study is based on self-reported behavioural data obtained from a non-probability sample of street-involved individuals. Consequently, there is potential for recall bias, socially desirable reporting, and misclassification of exposure variables in this study. Responses to questions concerning drug use and sexual behaviors may be influenced by the participant’s knowledge of their HCV antibody status. In addition, recall problems may exist with reporting of past traumatic life events. The effect of memory on our estimates of risk for these factors is difficult to assess. Additionally, although data were analyzed using time-dependent variables, we cannot make conclusions regarding temporal sequences between HCV risk patterns and infection. The number of participants who had never used injection drugs and seroconverted to HCV positive over the study period was very low (n = 5) we were therefore unable to examine the risks for HCV incidence among those participants. Finally, the fact that HCV incidence was lower among participants who had been injecting drugs longer may be demonstrating a survivorship bias. Therefore, perhaps the most at-risk participants who had been injecting longest became HCV positive prior to entering the study. Nevertheless, this study does show alarming rates of HCV infection early in the injection career of young Aboriginal people in Vancouver and Prince George. Despite these limitations, we believe these data provide important epidemiological information about a high-risk Aboriginal population that has not been previously studied.

## Conclusions

Results presented in this study suggest that even in the presence of established harm reduction initiatives in both Vancouver and Prince George, including fixed and mobile needle distribution sites, young Aboriginal people who use drugs are at sustained risk for HCV infection. In addition, the new initiates to injection drug use were at highest risk, particularly those young people who were frequently injecting cocaine, sharing rigs and involved in sex work. In the absence of post-exposure prophylaxis or an effective HCV vaccine, primary prevention programs must focus on safe injection practices and reducing the number of people who initiate injection drug use. Indeed, such high incident rates among new initiates in this study underscore the importance of carefully examining the effectiveness of current harm reduction initiatives in both rural and urban settings. On September 30, 2011, the Supreme Court of Canada made a unanimous decision ordering the federal government to refrain from its attempts to close Vancouver’s supervised injection clinic [29]. It has been speculated that this landmark decision will provide other Canadian cities with the authority to open their own safe injection clinics [30]. Although not statistically significant, the present study indicated a higher incidence of HCV in the northern city of Prince George than Vancouver. Based on the estimates we have obtained in this study, we speculate similar rates of HCV infection will be observed in smaller, northern communities throughout British Columbia. In previous studies, we identified Vancouver’s safe injection facility as an integral source for harm reduction programming among Cedar Project participants who were already fully entrenched in injection drug use and HCV positive [31]. Taken together, these findings underscore the urgent need for expansion of safe drug use facilities to other locations in BC, including the North. Furthermore, current policies and practices guiding addiction treatment delivery may be reinforcing the marginalization of street-involved young Aboriginal people because they fail to address historical and social injustices that influence resilience, particularly racial discrimination [32-34]. Treatment delivery for optimal adherence among young, HCV positive Aboriginal people who inject drugs must be individually tailored, enriched with ancillary psychosocial supports and provided within a culturally safe setting [7,35]. These findings may be applicable to at-risk young Indigenous people globally, for example in Australia, where similar patterns of vulnerabilities have been identified [36]. With significant increases in resources, acknowledging the intergenerational trauma related to the residential school system may be one way that Aboriginal leadership and addiction specialists and other practitioners can begin to mitigate the potential impact of the epidemic currently threatening their communities.

## Competing interests

The authors declare that they have no competing interests.

## Authors’ contributions

PMS wrote the initial draft of the manuscript was responsible for interpretation of the findings. MEP revised subsequent drafts of the manuscript and contributed to the statistical interpretation of the findings. AKM conducted the statistical analyses and helped draft the manuscript. NC, MT, WMC and MTS were responsible for the interpretation of the findings and revisions to the manuscript. All authors read and approved the final version of this manuscript.

## Funding

The study was supported by a grant from the Institute for Aboriginal Peoples Health, of the Canadian Institutes for Health Research (CIHR), which has no role in the preparation of data or manuscripts. PMS is the recipient of the CIHR New Investigator Career Award. MEP and NC are supported by CIHR doctoral research awards. WMC is the spokesperson for the Shuswap Nation Tribal Council. MTS is the Chief Scientific Officer for the Michael Smith Foundation for Health Research.

## Pre-publication history

The pre-publication history for this paper can be accessed here:

http://www.biomedcentral.com/1471-2458/12/632/prepub
